# Associations between tri-ponderal mass index, body mass index, and high blood pressure among children and adolescents: a cross-sectional study

**DOI:** 10.1038/s41598-023-45432-5

**Published:** 2023-10-24

**Authors:** Renata Kuciene, Virginija Dulskiene

**Affiliations:** Institute of Cardiology, Medical Academy, Lithuanian University of Health Sciences, Sukileliu 15, 50162 Kaunas, Lithuania

**Keywords:** Risk factors, Cardiology

## Abstract

High blood pressure (HBP) and obesity are major public health issues globally. The aim of the study was to evaluate the associations between tri-ponderal mass index (TMI) and body mass index (BMI) and HBP and to determine which anthropometric parameters may best predict HBP among Lithuanian children and adolescents aged 7–18 years. This cross-sectional study included 3710 Lithuanian children and adolescents aged 7–18 (52.7% boys and 47.3% girls). Each subject’s height, weight, and other anthropometric parameters, as well as blood pressure were measured according to standardized protocols; subsequently, TMI and BMI were calculated. The prevalence of HBP was 27% (the prevalence of elevated BP and hypertension was 13.7% and 13.3%, respectively), significantly higher for boys than for girls. The Pearson correlation coefficients between the BMI z-score and BP were higher than those between the TMI z-score and BP. In both sexes combined, the adjusted odds ratios (aOR) for HBP were increased significantly with increasing quartiles of TMI and BMI as compared to the first quartile (Q1) (Q2: aOR = 1.37 and aOR = 1.69; Q3: aOR = 2.10 and aOR = 2.27; Q4: aOR = 3.95 and aOR = 4.91, respectively). Significant associations also were observed between overweight and obesity (defined according to two methods: age- and sex-specific TMI percentiles and IOTF criteria) among boys and girls separately. BMI presented a higher area under the curve value than TMI for predicting HBP in children and adolescents. The findings of the study suggest that BMI and TMI are significantly associated with HBP. However, BMI is a better predictor for HBP than TMI among Lithuanian children and adolescents aged 7–18 years.

## Introduction

High blood pressure (HBP) is a considerable public health challenge and is becoming more prevalent among children and adolescents worldwide^1^. Hypertension in children and adolescents is associated with adverse subclinical cardiovascular outcomes, left ventricular hypertrophy, increased carotid intima-media thickness, higher pulse wave velocity, atherosclerotic changes, and retinal vascular changes^2^. HBP in childhood is significantly related to hypertension in adulthood^3^ and is a known risk factor for major adverse cardiovascular events, coronary heart disease, stroke, heart failure, and cardiovascular death later in life^4^. Early identification of HBP in children and adolescents may help prevent the development and progression of hypertension and its complications in adulthood.

The prevalence of obesity is increasing worldwide. In 2016, 74 million boys and 50 million girls aged 5–19 years had obesity^5^. Childhood obesity tends to persist into adolescence and then adulthood, and children with obesity, compared to normal-weight children, have a more than five times greater risk of becoming adults with obesity^6^. Obesity in children and adolescents is associated with many health complications and comorbidities, including hypertension, left ventricular hypertrophy, endothelial dysfunction, dyslipidaemia, type 2 diabetes, metabolic syndrome, impaired glucose tolerance, delayed or accelerated puberty, and musculoskeletal, gastrointestinal, psychological, and sleep disorders^7^.

In research settings and in clinical practice, BMI is a widely used measure for assessing the body weight status in children, adolescents, and adults. However, BMI cannot distinguish between body fat mass and lean mass^8^. Peterson et al.^9^ suggested TMI (mass divided by height cubed), the origin of which is based on the Ponderal index and the Rohrer Index, and also showed that this screening tool estimated body fat mass and overweight status more accurately than BMI among children and adolescents. A study by Wang et al.^10^ demonstrated that BMI performed marginally better than TMI for identifying dyslipidaemia for both sexes combined in both Chinese and American young populations. A systematic review and meta-analysis revealed that subjects with normal weight obesity—i.e., those having a normal body mass index, but a high-fat percentage—have increased odds of cardiometabolic risk factors compared to subjects with normal weight non-obesity^11^. Epidemiological studies have investigated the prediction of HBP by TMI and BMI among children and adolescents^10,12–16^, but the results were inconsistent. Some research studies have revealed that both TMI and BMI similarly predicted the paediatric metabolic syndrome^17^ or clustered cardio-metabolic risk factors^18^. Growing scientific evidence analysing the associations between TMI and BMI and cardiometabolic risk factors in children and adolescents has also been described in recent systematic review by Sun et al.^19^. An increased BMI is known to be associated with HBP in children and adolescents^20,21^. In addition, children with overweight or obesity have increased odds for other adverse cardiometabolic risk factors (higher levels of triglycerides, total cholesterol, and low-density lipoprotein cholesterol)^22^. However, there is a limited number of studies on the association between overweight and obesity defined by TMI and HBP^23,24^. The Korea National Health and Nutrition Examination Survey reported that youth aged 10–20 years in the overweight and obesity group (according to age- and sex-specific TMI) had an increased risk for cardiometabolic risk factors^23^.

In Lithuania, the prevalence of selected cardiovascular risk factors in paediatric population has increased over the last 2 decades. Epidemiological studies conducted in Lithuania have shown a high prevalence of HBP in preschool children aged 3–7 years (21.4%)^25^ and prehypertension and hypertension in adolescents aged 12–15 years (12.8% and 22.2%, respectively)^26^. The prevalence of overweight and obesity in adolescents aged 12–15 years was 12.1% and 2.4%, respectively^27^, while in children and adolescents aged 7–17 years, it was 12.6% and 4.1%, respectively^28^. Thus, in the present study, we aimed to investigate the associations of TMI and BMI with HBP and to determine which anthropometric parameter was the best predictor of HBP in Lithuanian children and adolescents.

## Results

The anthropometric characteristics and BP of the 3710 included subjects (52.7% boys) are presented in Table 1. The mean age was 10.91 ± 2.85 years. The mean BMI, weight, height, HC, MUAC, NC, WC, BSA, WHR, WHtR, SBP, and PP were significantly higher in boys than in girls. Girls had a significantly greater DBP compared to boys. There were no statistically significant differences in mean age, TMI, or MAP between the two groups. Mean BMI was higher for boys (18.80 ± 3.82) than for girls (18.50 ± 3.63) (*P* = 0.04), while mean TMI was higher for girls (12.52 ± 2.11) than for boys (12.47 ± 2.14) (*P* = 0.281).

**Table 1:** Demographic, anthropometric, and BP characteristics of the study participants by sex.

Variables	Total (n = 3710)	Boys (n = 1954)	Girls (n = 1756)	P*
Age (years)	10.91 ± 2.85	10.95 ± 2.85	10.87 ± 2.85	0.399
Weight (kg)	43.30 ± 16.35	44.68 ± 17.65	41.77 ± 14.63	< 0.001
Height (cm)	149.60 ± 17.21	151.13 ± 18.40	147.90 ± 15.60	< 0.001
BMI(kg/m^2^)	18.66 ± 3.74	18.80 ± 3.82	18.50 ± 3.63	0.04
TMI (kg/m^3^)	12.49 ± 2.13	12.47 ± 2.14	12.52 ± 2.11	0.281
HC (cm)	78.91 ± 11.50	79.43 ± 11.68	78.33 ± 11.28	0.009
MUAC (cm)	22.00 ± 3.20	22.19 ± 3.46	21.57 ± 2.99	< 0.001
NC (cm)	29.00 ± 3.05	29.76 ± 3.29	28.15 ± 2.51	< 0.001
WC (cm)	63.00 ± 9.99	64.75 ± 10.77	61.06 ± 8.66	< 0.001
BSA	1.33 ± 0.32	1.36 ± 0.34	1.30 ± 0.29	< 0.001
WHR	0.80 ± 0.07	0.82 ± 0.06	0.78 ± 0.07	< 0.001
WHtR	0.42 ± 0.05	0.43 ± 0.05	0.41 ± 0.05	< 0.001
SBP(mm Hg)	109.26 ± 13.54	110.50 ± 14.28	107.89 ± 12.54	< 0.001
DBP (mm Hg)	62.40 ± 8.17	61.89 ± 8.12	62.98 ± 8.20	< 0.001
MAP (mm Hg)	78.02 ± 8.62	78.09 ± 8.71	77.95 ± 8.52	0.889
PP (mm Hg)	46.86 ± 11.87	48.61 ± 12.72	44.91 ± 10.52	< 0.001

The values are mean ± SD.

*BMI* Body mass index, *TMI* Tri‑ponderal mass index, *HC* Hip circumference, *MUAC* Mid-upper arm circumference, *NC* Neck circumference, *WC* Waist circumference, *BSA* Body surface area, *WHR* Waist-hip ratio, *WHtR* Waist-to-height ratio, *SBP* Systolic blood pressure, *DBP* Diastolic blood pressure, *MAP* Mean arterial pressure, *PP* Pulse pressure.

* Boys versus girls.

Overall, the prevalence of HBP was 27.0% (29.7% for boys and 23.9% for girls) (Table 2). The prevalence of elevated BP and hypertension was 13.7% (14.5% for boys and 12.8% for girls) and 13.3% (15.2% for boys and 11.2% for girls), respectively. Thus, the prevalence of HBP was significantly higher in boys than in girls. Subjects aged 11–14 and 15–18 years were more likely to have HBP compared to those aged 7–10 years (OR = 2.55 and OR = 8.11, both *P* < 0.001, respectively). Subjects with HBP had significantly higher mean values of weight, height, BMI, TMI, HC, MUAC, NC, WC, BSA, WHtR, SBP, DBP, MAP, and PP, compared to normotensive subjects. In boys and girls separately, the mean value of WHR was higher in the normotensive group than in the HBP group, but in boys, no significant difference between these groups in the mean WHR was found. The overall prevalence of overweight and obesity using age- and sex-specific TMI percentiles was 10.2% and 4.8%, respectively. Among boys, 10.1% had overweight and 4.8% had obesity. Likewise, among girls, 10.4% had overweight and 4.9% had obesity. Based on the IOTF criteria, the prevalence of overweight and obesity was 16.3% and 5.8% (for boys: 17.7% and 6.9%; for girls: 14.7% and 4.7%), respectively.

**Table 2:** Characteristics of the study participants according to BP level.

Variables	Normal BP	HBP	P*
Boys			
Quartiles of TMI			< 0.001
1st	386 (28.2)	96 (16.5)*	
2nd	375 (27.3)	115 (19.8)*	
3rd	337 (24.5)	155 (26.7)	
4th	275 (20.0)	215 (37.0)*	
Quartiles of BMI			< 0.001
1st	397 (28.9)	85 (14.6)*	
2nd	363 (26.5)	120 (20.7)*	
3rd	341 (24.8)	157 (27.0)	
4th	272 (19.8)	219 (37.7)*	
TMI categories			< 0.001
Underweight/Normal weight	1223 (89.1)	441 (75.0)*	
Overweight	109 (7.9)	88 (16.0)*	
Obesity	41 (3.0)	52 (9.0)*	
BMI categories (IOTF)			< 0.001
Normal weight	1114 (81.1)	360 (62.0)*	
Overweight	203 (14.8)	143 (24.6)*	
Obesity	56 (4.1)	78 (13.4)*	
Weight (kg)	38.62 ± 13.32	59.02 ± 18.32	< 0.001
Height (cm)	145.51 ± 15.74	164.41 ± 17.39	< 0.001
BMI (kg/m^2^)	17.72 ± 3.10	21.35 ± 4.15	< 0.001
TMI (kg/m^3^)	12.22 ± 1.92	13.05 ± 2.48	< 0.001
HC (cm)	75.34 ± 9.58	89.09 ± 10.43	< 0.001
MUAC (cm)	21.01 ± 2.77	24.98 ± 3.31	< 0.001
NC (cm)	28.61 ± 2.57	32.47 ± 3.20	< 0.001
WC (cm)	61.39 ± 8.67	72.68 ± 11.07	< 0.001
BSA	1.24 ± 0.27	1.63 ± 0.33	< 0.001
WHR	0.82 ± 0.06	0.81 ± 0.06	0.153
WHtR	0.42 ± 0.05	0.44 ± 0.06	< 0.001
SBP(mm Hg)	103.44 ± 8.93	127.17 ± 10.10	< 0.001
DBP (mm Hg)	59.61 ± 6.89	67.27 ± 8.25	< 0.001
MAP (mm Hg)	74.22 ± 6.26	87.24 ± 6.62	< 0.001
PP (mm Hg)	43.83 ± 9.26	59.90 ± 12.66	< 0.001
Girls			
Quartiles of TMI			< 0.001
1st	375 (28.1)	61 (14.6)*	
2nd	352 (26.3)	85 (20.2)*	
3rd	333 (24.9)	113 (26.9)	
4th	276 (20.7)	161 (38.3)*	
Quartiles of BMI			< 0.001
1st	377 (28.2)	57 (13.6)*	
2nd	349 (26.1)	89 (21.2)*	
3rd	345 (25.8)	101 (24.0)	
4th	265 (19.9)	173 (41.2)*	
TMI categories			< 0.001
Underweight/Normal weight	1182 (88.5)	306 (72.9)*	
Overweight	118 (8.8)	64 (15.2)*	
Obesity	36 (2.7)	50 (11.9)*	
BMI categories (IOTF)			< 0.001
Normal weight	1134 (84.9)	282 (67.1)*	
Overweight	162 (12.1)	96 (22.9)*	
Obesity	40 (3.0)	42 (10.0)*	
Weight (kg)	39.09 ± 13.17	50.28 ± 15.74	< 0.001
Height (cm)	145.86 ± 15.36	154.36 ± 14.60	< 0.001
BMI (kg/m^2^)	17.83 ± 3.14	20.63 ± 4.22	< 0.001
TMI (kg/m^3^)	12.25 ± 1.87	13.38 ± 2.56	< 0.001
HC (cm)	76.19 ± 10.36	85.12 ± 11.42	< 0.001
MUAC (cm)	21.03 ± 2.72	23.28 ± 3.15	< 0.001
NC (cm)	27.72 ± 2.34	29.50 ± 2.53	< 0.001
WC (cm)	59.50 ± 7.58	66.01 ± 9.94	< 0.001
BSA	1.25 ± 0.27	1.46 ± 0.29	< 0.001
WHR	0.79 ± 0.07	0.78 ± 0.07	0.020
WHtR	0.41 ± 0.05	0.43 ± 0.06	< 0.001
SBP mm Hg)	103.23 ± 9.02	122.70 ± 10.51	< 0.001
DBP (mm Hg)	60.64 ± 6.93	70.40 ± 7.47	< 0.001
MAP (mm Hg)	74.84 ± 6.55	87.83 ± 6.24	< 0.001
PP (mm Hg)	42.59 ± 8.56	52.30 ± 12.56	< 0.001
Boys			
Age (years)			< 0.001
7–10	825 (60.1)	139 (23.9)*	
11–14	483 (35.2)	251 (43.2)*	
15–18	65 (4.7)	191 (32.9)*	
Age (years)	10.11 ± 2.39	12.94 ± 2.86	< 0.001
Girls			
Age (years)			< 0.001
7–10	739 (55.3)	143 (34.0)*	
11–14	468 (35.0)	186 (44.3)*	
15–18	129 (9.7)	91 (21.7)*	
Age (years)	10.51 ± 2.74	12.01 ± 2.89	< 0.001

The values are numbers (percentages) and mean ± SD (standard deviation).

The chi-squared (χ^2^) test was used for categorical variables.

*BP* Blood pressure, *BMI* Body mass index, *TMI* Tri‑ponderal mass index, *HC* Hip circumference, *MUAC* Mid-upper arm circumference, *NC* Neck circumference, *WC* Waist circumference, *BSA* Body surface area, *WHR* Waist-hip ratio, *WHtR* Waist-to-height ratio, *SBP* Systolic blood pressure, *DBP* Diastolic blood pressure, *MAP* Mean arterial pressure, *PP* Pulse pressure.

* *P* < 0.05 versus NBP group (z test).

The prevalence of HBP increased with increasing TMI (Supplementary Table 3) and BMI (Supplementary Table 4) quartiles in both sexes (*P* < 0.001): TMI: 4.9%, 5.9%, 7.9%, and 11.0% for boys and 3.5%, 4.8%, 6.4%, and 9.2% for girls; BMI: 4.4%, 6.1% 8.0%, and 11.2% for boys and 3.2%, 5.1%, 5.8%, and 9.9% for girls. Boys had a higher prevalence of HBP than girls across increasing quartiles of TMI and BMI (*P* < 0.001). In both sexes, the mean values of anthropometric variables including weight, WC, HC, NC, MUAC, BSA, WHtR, WHR, and the mean values of BP increased with increasing TMI and BMI quartiles, while the mean values of age and height were not significantly different across the quartiles of TMI. The subjects with obesity defined according to age- and sex-specific TMI percentiles (Supplementary Table 5) and the IOTF criteria (Supplementary Table 6) had the highest values of BP compared to their counterparts with normal weight.

The TMI z-score and BMI z-score significantly and positively correlated with SBP, DBP, MAP, and PP in boys and in girls, but the BMI z-score correlated more strongly with all BP measurements than the TMI z-score did (Table 3). The correlations of the TMI z-score with all BP parameters and the correlation of the BMI z-score with DBP in girls were slightly higher than in boys. The highest correlation coefficients were defined for the BMI z-score and SBP (in boys: r = 0.557; in girls: r = 0.503) and MAP (in boys: r = 0.511; in girls: r = 0.492).

**Table 3:** Pearson’s correlation coefficients between anthropometric parameter z-scores and blood pressure.

	TMI z-score	BMI z-score
SBP (mm Hg)		
Boys	0.188**	0.557**
Girls	0.247**	0.503**
Total	0.211**	0.534**
DBP (mm Hg)		
Boys	0.130**	0.333**
Girls	0.182**	0.382**
Total	0.155**	0.352**
MAP (mm Hg)		
Boys	0.184**	0.511**
Girls	0.238**	0.492**
Total	0.209**	0.502**
PP (mm Hg)		
Boys	0.128**	0.412**
Girls	0.153**	0.302**
Total	0.134**	0.368**

*TMI* Tri‑ponderal mass index, *BMI* Body mass index, *SBP* Systolic blood pressure, *DBP* Diastolic blood pressure, *MAP* Mean arterial pressure, *PP* Pulse pressure.

**Correlation is significant at the level of 0.01 (2-tailed).

The BMI z-score correlated significantly with weight (for boys: r = 0.844; for girls: r = 0.860; all *P* values were < 0.001), height (for boys: r = 0.512; for girls: r = 0.507; all *P* values were < 0.001), and WC (for boys: r = 0.849; for girls: r = 0.834; all *P* values were < 0.001). The TMI z-score positively and significantly correlated with weight (for boys: r = 0.363; for girls: r = 0.446; all *P* values were < 0.001) and WC (for boys: r = 0.539; for girls: r = 0.637; all *P* values were < 0.001) and significantly and negatively correlated with height (for boys: r = -− 0.093; for girls: r = − 0.038; all *P* values were < 0.001).

The mean values of the TMI according to age and sex were fairly stable, ranging from 12.05 to 12.72 kg/m^3^ for boys and 12.14 to 13.11 kg/m^3^ for girls. The 50th percentile values for the TMI ranged from 11.44 to 12.49 kg/m^3^ for boys and 11.72 to 12.76 kg/m^3^ for girls. The 85th percentile values ranged from 13.63 to 15.27 kg/m^3^ for boys and 13.97 to 15.04 kg/m^3^ for girls. The 95th percentile values ranged from 15.59 to 17.83 kg/m^3^ for boys and 15.73 to 17.61 kg/m^3^ for girls (Supplementary Table 1; Supplementary Fig. 1).

In the multivariate logistic regression analysis, after adjustment for age and sex, in both sexes combined, the aORs for HBP were 1.37 for the second quartile of the TMI, 2.10 for the third quartile, and 3.95 for the fourth quartile as compared with the first quartile (all *P* < 0.05) (Table 4). Correspondently, aORs for the quartiles of the BMI were 1.69, 2.27, and 4.91 (all *P* < 0.05). Similar associations were found in girls. In boys, no significant association was observed for HBP in the second quartile of the TMI.

**Table 4:** Associations between the categories of anthropometric parameters and the risk of HBP (univariate and multivariate analyses).

Variables	Boys		Girls		Total	
	OR (95% CI)	aOR^1^ (95% CI)	OR (95% CI)	aOR^1^ (95% CI)	OR (95% CI)	aOR^2^ (95% CI)
Quartiles of TMI						
1st	1.00	1.00	1.00	1.00	1.00	1.00
2nd	1.23 (0.91–1.67)^NS^	1.29 (0.91–1.81)^NS^	1.48 (1.04–2.13)*	1.51 (1.04–2.18)*	1.33 (1.06–1.68)*	1.37 (1.07–1.76)*
3rd	1.85 (1.38–2.48)	2.14 (1.54–2.98)	2.09 (1.48–2.94)	2.14 (1.51–3.05)	1.94 (1.55–2.42)	2.10 (1.65–2.66)
4th	3.14 (2.36–4.18)	4.31 (3.10–5.97)	3.59 (2.57–5.00)	3.83 (2.72–5.40)	3.31 (2.67–4.11)	3.95 (3.13–4.99)
Quartiles of BMI						
1st	1.00	1.00	1.00	1.00	1.00	1.00
2nd	1.54 (1.13–2.11)*	1.70 (1.20–2.41)*	1.69 (1.17–2.43)*	1.72 (1.19–2.49)*	1.60 (1.26–2.03)	1.69 (1.31–2.17)
3rd	2.15 (1.59–2.91)	2.65 (1.89–3.72)	1.94 (1.36–2.76)	1.99 (1.39–2.87)	2.05 (1.63–2.58)	2.27 (1.77–2.89)
4th	3.76 (2.80–5.05)	5.44 (3.88–7.62)	4.32 (3.08–6.06)	4.68 (3.31–6.63)	3.98 (3.19–4.96)	4.91 (3.87–6.24)
TMI categories						
Underweight/Normal weight	1.00	1.00	1.00	1.00	1.00	1.00
Overweight	2.24 (1.66–3.03)	2.73 (1.94–3.84)	2.10 (1.51–2.91)	2.09 (1.49–2.94)	2.16 (1.73–2.69)	2.34 (1.84–2.97)
Obesity	3.52 (2.30–5.37)	5.00 (3.14–7.97)	5.37 (3.43–8.38)	5.90 (3.72–9.34)	4.27 (3.14–5.80)	5.37 (3.87–7.46)
BMI categories (IOTF)						
Normal weight	1.00	1.00	1.00	1.00	1.00	1.00
Overweight	2.18 (1.71–2.78)	2.59 (1.96–3.43)	2.38 (1.79–3.17)	2.60 (1.94–3.50)	2.29 (1.91–2.76)	2.58 (2.11–3.16)
Obesity	4.31 (3.00–6.20)	5.91 (3.94–8.86)	4.22 (2.67–6.64)	5.44 (3.41–8.68)	4.38 (3.30–5.81)	5.71 (4.22–7.74)

TMI, tri‑ponderal mass index; BMI, body mass index; OR, odds ratio; aOR^1^, adjusted odds ratio for age; aOR^2^, adjusted odds ratio for age and sex; CI, confidence interval.

All results were significant at *P* < 0.0001, except when noted (NS – not significant; * – *P* < 0.05).

After adjustment for age, statistically significant elevated aORs were observed for associations between overweight and obesity (based on age- and sex-specific TMI percentiles) and HBP in both sexes (among boys: aOR = 2.73 and aOR = 5.00, respectively, and among girls: aOR = 2.09 and aOR = 5.90, respectively, (all *P* < 0.001)). Overweight and obesity (defined by the IOTF criteria) also were associated with HBP (among boys: aOR = 2.59 and aOR = 5.91, respectively, and among girls: aOR = 2.60 and aOR = 5.44, respectively, (all *P* < 0.001)). The results of both methods also remained statistically significant in both sexes combined.

Table 5 shows the results of the ROC analysis for BMI and TMI for the prediction of HBP in boys and girls separately. BMI presented higher area under the curve (AUC) values than TMI for predicting HBP (all AUC values were *P* < 0.001) in both sexes. The highest AUCs were found for BMI in 7–10 year-old (AUC = 0.713) and 11–14 year-old boys (AUC = 0.724). The AUC values of BMI and TMI were greater in boys than in girls, except for 15- to 18-year-old adolescents (Supplementary Fig. 3).

**Table 5:** Area under ROC curves (95% CI) of anthropometric indices to predict HBP.

	TMI				BMI			
	AUC	(95% CI)	SE	*P* value	AUC	(95% CI)	SE	*P* value
Boys								
7–10	0.664	0.614–0.713	0.025	< 0.001	0.713	0.667–0.758	0.023	< 0.001
11–14	0.650	0.608–0.693	0.022	< 0.001	0.724	0.687–0.762	0.019	< 0.001
15–18	0.648	0.575–0.721	0.037	< 0.001	0.663	0.590–0.737	0.038	< 0.001
Girls								
7–10	0.649	0.598–0.699	0.026	< 0.001	0.672	0.623–0.721	0.025	< 0.001
11–14	0.617	0.568–0.666	0.025	< 0.001	0.656	0.609–0.703	0.024	< 0.001
15–18	0.679	0.607–0.751	0.037	< 0.001	0.686	0.614–0.757	0.036	< 0.001

Data are AUC (95% confidence interval).

*AUC* Area under the receiver operating characteristic curve, *TMI* Tri‑ponderal mass index, *BMI* Body mass index, *SE* standard error.

## Discussion

To our knowledge, this is the first cross-sectional study in Lithuania to investigate the association between TMI and HBP, and, also, to identify which of the anthropometric indices (TMI or BMI) can best predict HBP in children and adolescent populations. In the present study, the subjects with HBP had higher mean TMI and BMI than those with normal BP did. Moreover, BMI and TMI significantly correlated with SBP, DBP, MAP, and PP. However, the correlations of TMI with BP were weaker than the correlations of BMI, and our findings are consistent with those of previous epidemiological studies conducted among children. The CASPIAN-V study carried out in Iranian children and adolescents suggested that BMI correlated with SBP and DBP more strongly than TMI did in both boys and girls in all age groups^17^. The infancy-onset study from Finland documented that BMI correlated significantly more strongly with SBP (age 5–20 years) than TMI did^15^.

Epidemiological studies demonstrated that TMI values were relatively stable from childhood to adolescence^9,18,29,30^. Numerous countries, including Korea^23^, Canada^12^, Brazil^24^, China^31^, Turkey^32^, and Iran^33^ have published national age- and sex-specific TMI percentile values for various age groups. However, there are no international agreements, standards, or recommendations for accepted TMI cut-off values which vary depending on age, sex, race, and ethnicity for defining overweight and obesity among children and adolescents. In the current study and also in other research studies^23,24^, the subjects with TMI values between ≥ 85th percentile and < 95th percentiles were considered as having overweight, and subjects with TMI values ≥ 95th percentile were considered as having obesity*.* However, the age- and sex-specific TMI cut-offs values to determine overweight and obesity differ in different countries and across studies of different paediatric populations. Using the KNHANES data, Shim reported that the 85th percentile values ranged from 14.67 to 15.73 kg/m^3^ for boys and 14.27 to 15.50 kg/m^3^ for girls, and the 95th percentile values ranged from 16.62 to 17.40 kg/m^3^ for boys and 16.00 to 17.72 kg/m^3^ for girls aged 10–18 years^23^. In a cross-sectional study in Brazilian adolescents aged 12–17 years, the 85th percentile values of TMI ranged between 14.3 to 15.7 kg/m^3^ in boys, and 15.3 to 16.5 kg/m^3^ in girls, and the 95th percentile values ranged from 16.8 to 17.7 kg/m^3^ for boys and 17.6 to 19.3 kg/m^3^ for girls^24^. According to the DAMTCA-II data, in boys, the TMI cut-off values for assessing adiposity and obesity were 14.9 kg/m^3^ (85th percentile) for children aged ≤ 12 years and 14.5 kg/m^3^ (75th percentile) for adolescents aged > 12 years, while in girls, the respective values were 15.8 kg/m^3^ (90th percentile) for children aged ≤ 10 years and 16.2 kg/m^3^ (95th percentile) for children aged > 10 years^32^. In a cross-sectional study using data from the NHANES, the TMI threshold values to define overweight were 16.0 kg/m^3^ for boys and 16.8 kg/m^3^ for girls, and the values to define obesity were 18.8 kg/m^3^ for boys and 19.7 kg/m^3^ for girls aged 8–17 years. However, age- and sex-specific TMI percentiles were not used to define the weight status category^9^.

In the current study, the prevalence of overweight and obesity determined according to the TMI percentiles in the subjects was lower than the prevalence of overweight and obesity determined according to the IOTF cut-offs, and these findings are consistent with those of a study on Italian adolescents^14^. In the Canadian Health Measures Survey, the prevalence of overweight defined by the TMI was lower than that defined by the IOTF BMI cut-offs (15% vs. 18%), but the prevalence of obesity defined by the TMI was slightly higher than that defined by the BMI (9.7% vs. 8.9%)^12^. In KNHANES, the prevalence of overweight defined by the TMI (≥ 85th percentile and < 95th percentile) was slightly higher than that defined by the BMI (≥ 85th percentile and < 95th percentile) (10.6% vs. 10.2%), but the prevalence of obesity defined by the TMI (≥ 95th percentile) was lower than that defined by the BMI (≥ 95th percentile) (5.3% vs. 10.6%) in children and adolescents aged 10–20 years^29^. In our study, children and adolescents with overweight and obesity classified according to age- and sex-specific TMI percentiles had higher mean values of BMI, TMI, WC, and BP compared to subjects with normal weight, which is in line with the results of other studies conducted in Brazilian adolescents^24^ and Korean youth^23^.

Multivariate logistic regression analysis of our data showed significant associations between overweight and obesity based on age- and sex-specific TMI percentiles and defined by the IOTF criteria and HBP in both sexes separately and combined. Furthermore, higher aORs for HBP were also found in subjects in the highest quartiles of TMI and BMI than in those within lower quartiles. The aORs for HBP in BMI quartiles were higher than in TMI quartiles in both sexes combined. Our findings are in line with those obtained in another previous study that reported significant associations of obesity defined according to age and sex-specific TMI with HBP^23^. In a Korean national survey, the aORs for elevated BP among subjects aged 10–20 years with obesity (based on age- and sex-specific TMI percentiles) were higher (aOR = 2.22) than among participants with overweight (aOR = 1.33) compared to those in the normal weight group. However, overweight was not associated with an elevated BP among girls^23^. Wang et al. observed that the TMI was significantly associated with HBP in Chinese (aOR 1.23) and American paediatric populations (aOR ranged from 1.16 to 1.17), whereas the corresponding ORs for BMI were slightly lower^10^. In a study on Chinese children aged 3–17 years, aORs of the TMI for hypertension (aORs ranged from 1.19 to 1.24) were greater than aORs of the BMI (aORs ranged from 1.14 to 1.17)^18^. A study on Brazilian adolescents carried out by Alvim et al. suggested that boys and girls with overweight and obesity according to the TMI percentiles had higher prevalence ratios for hypertension, insulin resistance, hypercholesterolemia, and hypertriacylglycerolemia, compared to subjects with normal weight^24^.

In the present study, the ROC analysis showed that the BMI had greater AUC values (ranged from 0.663 to 0.724 for boys and from 0.656 to 0.672 for girls) than the TMI did and was also a better predictor of HBP, while the TMI had a slightly lower AUC values (ranged from 0.648–0.664 for boys and from 0.617 to 0.679 for girls). Prior studies that examined the predictive ability of the TMI and the BMI for HBP revealed mixed findings. For instance, according to data from a 20-year infancy-onset cohort in Finland, the AUC values of the BMI were significantly higher than those of the TMI for hypertension (difference in AUC = 0.017–0.022) among youth aged 16–20 years, and the BMI was superior to the TMI in predicting obesity-related outcomes in adulthood^15^. A monocentric and retrospective cross-sectional study among Italian adolescents aged 12–13 years found that the TMI was better than the BMI for discriminating hypertension in both sexes (AUC values for boys: TMI 0.73 and BMI 0.70; and AUC values for girls: TMI 0.76 and BMI 0.73)^14^. Chen et al. demonstrated that the AUC of the TMI was significantly higher than that of the BMI for identifying hypertension in Chinese children aged 3–17 years (0.64 and 0.61)^18^. A cross-sectional study including Caucasian children and adolescents with obesity observed the strongest association between the BMI z-score and HBP (the lowest AIC value) among participants aged < 10 years. Furthermore, the BMI z-score was also the best predictor of HBP in girls aged ≥ 10 years, while the TMI better predicted HBP in boys aged ≥ 10 years^13^. The results of a Chinese national school-based health survey and the NHANES revealed that for Chinese children aged 7–18 years, the AUC values of the TMI (0.668 for boys and 0.602 for girls) was higher than that of the BMI (0.605 for boys and 0.537 for girls), whereas for American adolescents aged 12–18 years, the BMI (0.699 for boys and 0.708 for girls) was better than the TMI (0.695 for boys and 0.704 for girls) in predicting HBP^10^. Hu et al. reported that the TMI may have a stronger predictive capacity than the BMI for hypertension, especially in girls and older adolescents (≥ 16 years of age)^16^. Ashley-Martin et al. found that the TMI was similar to the BMI z-score in predicting HBP (SBP: 0.66 and 0.66; DBP: 0.56 and 0.55) in Canadian children and youth aged 6–19 years^12^.

The associations between the TMI and the BMI and obesity-related cardiovascular risk factors have been studied in children and adolescents. Also, attempts have been made to identify the best predictor of the metabolic syndrome; however, the findings differed among studies^19^. Several studies showed that the BMI was a better screening tool than the TMI in detecting the metabolic syndrome in Italian^34^ and Portuguese^35^ children and adolescents aged 10–17 years. The findings of the study conducted in Brazil showed that the BMI presented a slightly higher capacity compared to the TMI to predict insulin resistance in both sexes combined in adolescents with overweight^36^. Malavazos et al. suggested that the TMI is a superior body fat index and it better discriminates central obesity among adolescents than the BMI does^14^. Another study found that the TMI more strongly correlated with fat mass percentage measured by dual-energy X-ray absorptiometry (DEXA) and central obesity compared to the BMI z-score in adolescents with type 2 diabetes mellitus^37^. The results of a prospective study showed that patients aged 8–18 years with metabolically unhealthy obesity had greater BMI, TMI, and WC, had higher values of triglycerides, BP, and HOMA-IR and a lower level of high-density lipoprotein cholesterol compared to patients with metabolically healthy obesity^38^.

The mechanisms underlying the association between obesity and hypertension are complex and remain unclear and include activation of the sympathetic nervous system and the renin–angiotensin–aldosterone system, hyperinsulinaemia, oxidative stress, inflammation, endothelial dysfunction, disturbed sodium homeostasis, vascular damage, and the abnormal levels of adipokines^39,40^. Interactions between genes and environmental factors also play an important role in the development of obesity and cardiovascular diseases^41^.

The strength of our study is the database covering a wide age range of Lithuanian children and adolescents aged 7–18 years. This is the first cross‐sectional school‐based survey to assess the associations of the TMI and HBP in Lithuanian schoolchildren. However, our study has limitations. The study was carried out only in one district; however, Kaunas district is the second largest district by population size in Lithuania. This was a cross-sectional study, and therefore a causal relationship cannot be confirmed. Skinfold measurements were not performed in this study. Blood biochemical parameters, including blood lipids, blood glucose, etc. were not examined. No information was collected on the pubertal status of the participants. A limited number of older schoolchildren aged 15–18 years participated in the study compared to the number of younger children. The validation of the diagnosis of HBP was based on the measurements of BP (average of three BP readings) at one visit on the day of our study, though the elevated BP measurements obtained by oscillometric devices should be repeated by auscultation on separate occasions to confirm the diagnosis^42^. Thus, the possibility that errors may have occurred in the classification of the subjects according to their BP status cannot be ruled out. Measuring BP at one visit may overestimate the prevalence of HBP; nonetheless, two or three measurements of BP during a single visit are frequently performed in epidemiological studies. Differences in sample sizes, the age of the examined children and adolescents, the measurement methodologies, different definitions for overweight and obesity in children and adolescents have also made it difficult to make comparisons across studies.

Anthropometry is a simple, quick, economical, non-invasive, and accurate method that is easy to use in research and clinical settings and it is useful as an initial screening tool to identify subjects with an increased cardiometabolic risk^43^. Definitely, public health policies and strategies in Lithuania should focus more on the understanding and prevention of chronic non-communicable diseases and the promotion of a healthy lifestyle among young people. Thus, it is essential to identify children and adolescents with HBP and overweight/obesity to reduce or prevent adverse cardiometabolic health outcomes.

## Conclusions

Increased TMI and BMI, specifically overweight and obesity, were associated with greater odds of HBP in Lithuanian children and adolescents. Among them, the BMI was a better predictor of HBP than the TMI in both boys and girls. More large-scale international and multi-ethnic research is needed to obtain more comprehensive results and to confirm these findings.

## Methods

### Study population

This cross-sectional study was performed in Kaunas district, which is the second largest district in Lithuania, from November 2019 to 16 March 2020. A total of 3757 participants (from the 1st through the 12th grade of all 29 participating schools; ages 7–18 years) were selected using a stratified two-stage cluster sampling design. All the invited primary schools, basic schools, pre-gymnasiums, and gymnasiums agreed to participate in the survey. Of 3757 schoolchildren, 47 were excluded owing to missing anthropometric data (weight and/or height). Thus, a total of 3710 subjects were included in the final statistical analysis.

The study was conducted according to the principles and standards of the Declaration of Helsinki. Kaunas Regional Ethics Committee for Biomedical Research at the Lithuanian University of Health Sciences approved the study (10 June 2019; protocol No. BE-2-42). Before the investigation, a written informed consent for participation was obtained from each participant’s parent or guardian and from all participants.

### Blood pressure measurements

Both BP and anthropometric measurements were performed at the subjects’ schools by a team of trained professionals: physicians, biomedical scientists, qualified laboratory assistants, and technicians according to a standardized protocol.

Three blood pressure measurements were obtained using an automatic BP monitor (OMRON M6; OMRON HEALTHCARE CO., LTD, Kyoto, Japan) with 5-min rest intervals. BP was measured in a sitting position after the subjects had 10 min of seated rest. An average of these three measurements was calculated and used in the analysis. The measurements were taken in the morning by a physician not wearing a white coat. In the morning, the day before the test day, the participants were advised to avoid consuming caffeine, coffee, green or black tea, energy drinks, and physical exercises. Mean arterial pressure (MAP) was calculated as: (SBP + (2 × DBP))/3^44^. Pulse pressure (PP) was calculated as the difference between SBP and DBP. According to the 2017 American Academy of Pediatrics Clinical Practice Guideline for Screening and Management of High Blood Pressure in Children and Adolescents^45,46^, for children aged < 13 years, normal BP was defined as BP < 90th percentile for age, sex, and height; elevated BP—as ≥ 90th percentile to < 95th percentile for age, sex, and height, and hypertension—as ≥ 95th percentile for age, sex, and height. For adolescents aged ≥ 13 years, normal BP was defined as < 120/ < 80 mm Hg; elevated BP—as 120/ < 80 to 129/ < 80 mm Hg; and hypertension—as BP ≥ 130/ ≥ 80 mm Hg. HBP for children aged 7–12 years and adolescents aged 13 to 17 years was defined as having an elevated BP or hypertension.

### Anthropometric measurements

The body weight of the participants, wearing light dresses and barefoot, was measured with OMRON BF511 body composition monitor to the nearest 0.1 kg. The height of the participants, without shoes, was measured using the Leicester height measure stadiometer (Marsden, HM-250P) to the nearest 0.1 cm. The tri-ponderal mass index (TMI) was calculated as the weight in kilograms divided by the height in metres cubed (kg/m^3^)^9^. The body mass index (BMI) was calculated as weight in kilograms divided by height in metres squared (kg/m^2^).

Mid-upper arm circumference (MUAC) was measured to the nearest 0.1 cm using a non-elastic flexible measuring tape, at the midpoint between the olecranon process and the acromion process. Waist circumference (WC) was measured in standing position, at the mid-point between the lower margin of the last palpable rib and the top of the iliac crest to the nearest 0.5 cm. Neck circumference (NC) was measured at the level of the thyroid cartilage, with the participants in the standing position and the head held erect, eyes facing forward. Hip circumference (HC) was measured over light clothing at the maximum circumference around the buttocks. NC and HC were measured with a non-elastic flexible measuring tape to the nearest of 0.5 cm. Waist-to-height ratio (WHtR) was calculated as WC (cm)/height (cm). Waist-to-hip ratio (WHR) was calculated as WC (cm)/HC (cm).

The body surface area (BSA) was calculated using the following formula^47^:

$$ BSA = \sqrt {\frac{{Height\left( {cm} \right) \times Weight\left( {kg} \right)}}{3600}} . $$

### Statistical analysis

Statistical data analysis was performed using IBM SPSS for Windows, version 27.0 (IBM, Armonk, NY, USA). *P* values of less than 0.05 were considered statistically significant. The categorical variables were presented as number and percentages and were compared using the chi-squared test and z-test. The Kolmogorov–Smirnov test was used to test the normality of distribution of continuous variables. The normally distributed continuous variables were presented as means and standard deviations (SD). The age- and sex-specific 3rd, 5th, 10th, 15th, 25th, 50th, 75th, 85th, 90th, 95th, and 97th percentile values for TMI (Supplementary Table 1; Supplementary Fig. 1) and BMI (Supplementary Table 2; Supplementary Fig. 2) were calculated. The subjects were divided into four groups according to quartiles of TMI and BMI. The subjects were also classified into four groups according to age- and sex-specific TMI percentiles: underweight (TMI was < 3rd percentile), normal weight (TMI was ≥ 3rd percentile and < 85th percentile), overweight (TMI was ≥ 85th percentile and < 95th percentile), and obesity (TMI was ≥ 95th percentile). Overweight and obesity were also defined using age- and sex- specific BMI cut-off point classification established by the IOTF (International Obesity Task Force)^48^.

Pearson’s correlation coefficients were calculated between anthropometric parameters (TMI z-score and BMI z-score) and BP. Univariate and multivariate logistic regression analyses were conducted separately for boys and girls, and for both sexes combined to evaluate the associations between the quartiles of TMI and BMI, overweight and obesity defined according to age- and sex-specific TMI percentiles, and the IOTF criteria^48^ and HBP. Receiver operating characteristic (ROC) curve analysis was performed and area under the curves (AUC) of TMI and BMI for predicting HBP were calculated. The AUC was considered as failed (0.5–0.6), poor (0.6–0.7), fair (0.7–0.8), good (0.8–0.9), or excellent (0.9–1)^49^.

### Supplementary Information

Supplementary Information.

## Data Availability

According to the Statute of the Lithuanian University of Health Sciences, the authors cannot share the data underlying this study. For inquires on the data, researchers should first contact the owner of the database, the Lithuanian University of Health Sciences.
