# Supplementary material for: Associations between tri-ponderal mass index, body mass index, and high blood pressure among children and adolescents: a cross-sectional study
Source: Sci Rep. 2023 Oct 24;13:18148. doi: 10.1038/s41598-023-45432-5 (PMC10598122; doi:10.1038/s41598-023-45432-5)
Supplement: Supplementary file 1 — Supplementary Information. [file 41598_2023_45432_MOESM1_ESM.docx]

**Supplementary information**

**Associations between tri-ponderal mass index, body mass index, and high blood pressure among children and adolescents: a cross-sectional study**

Renata Kuciene^*^, Virginija Dulskiene

*Corresponding author:

Renata Kuciene – Institute of Cardiology, Medical Academy, Lithuanian University of Health Sciences; Sukileliu 15, LT-50162, Kaunas, Lithuania

e-mail: [renata.kuciene@lsmuni.lt](mailto:renata.kuciene@lsmuni.lt)

Virginija Dulskiene – Institute of Cardiology, Medical Academy, Lithuanian University of Health Sciences; Sukileliu 15, LT-50162, Kaunas, Lithuania

e-mail: [virginija.dulskiene@lsmuni.lt](mailto:virginija.dulskiene@lsmuni.lt)

**Supplementary Table 1.** Age- and sex-specific percentile values of the TMI in study participants aged 7–18 years.

| **Age** | **Mean ± SD** | | **Percentiles** | | | | | | | | | | |
| --- | --- | --- | --- | --- | --- | --- | --- | --- | --- | --- | --- | --- | --- |
|  |  |  | 3rd | 5th | 10th | 15th | 25th | 50th | 75th | 85th | 90th | 95th | 97th |
| ***Boys*** |  |  |  |  |  |  |  |  |  |  |  |  |  |
| 7 | 12.72±1.68 | | 10.38 | 10.47 | 10.71 | 10.99 | 11.58 | 12.49 | 13.60 | 14.47 | 14.88 | 15.59 | 16.63 |
| 8 | 12.49±2.10 | | 9.78 | 10.13 | 10.48 | 10.75 | 11.19 | 12.04 | 13.43 | 14.31 | 14.98 | 16.86 | 17.50 |
| 9 | 12.62±1.80 | | 10.17 | 10.43 | 10.74 | 10.93 | 11.25 | 12.22 | 13.62 | 14.37 | 14.81 | 16.36 | 16.96 |
| 10 | 12.60±2.16 | | 9.55 | 9.84 | 10.13 | 10.57 | 11.15 | 12.13 | 13.78 | 14.81 | 15.42 | 16.81 | 17.53 |
| 11 | 12.53±2.48 | | 9.23 | 9.40 | 9.99 | 10.26 | 10.89 | 12.02 | 13.68 | 14.95 | 15.99 | 17.83 | 18.25 |
| 12 | 12.45±2.18 | | 9.47 | 9.65 | 10.11 | 10.40 | 10.71 | 11.96 | 13.55 | 14.89 | 15.67 | 16.82 | 17.87 |
| 13 | 12.38±2.44 | | 9.01 | 9.26 | 9.71 | 10.12 | 10.74 | 11.73 | 13.81 | 15.27 | 15.81 | 17.34 | 17.82 |
| 14 | 12.29±2.30 | | 9.29 | 9.35 | 9.79 | 10.05 | 10.71 | 11.75 | 13.50 | 14.87 | 15.51 | 17.16 | 18.13 |
| 15 | 12.05±1.74 | | 9.71 | 9.86 | 10.33 | 10.57 | 10.79 | 11.57 | 13.02 | 14.17 | 15.94 | 16.64 | 17.65 |
| 16 | 12.07±2.22 | | 9.23 | 9.46 | 10.11 | 10.29 | 10.72 | 11.44 | 12.93 | 13.91 | 14.70 | 17.01 | 18.86 |
| 17 | 12.24±2.12 | | 9.45 | 9.78 | 9.97 | 10.17 | 10.81 | 11.88 | 13.16 | 13.63 | 15.16 | 16.78 | 18.31 |
| 18 | 12.26±2.20 | | 9.67 | 9.79 | 9.95 | 10.25 | 10.70 | 11.91 | 13.20 | 13.75 | 15.02 | 16.67 | 18.96 |
| ***Girls*** | | | | | | | | | | | | | |
| 7 | 12.91±2.18 | | 9.76 | 10.23 | 10.75 | 11.05 | 11.56 | 12.52 | 13.79 | 14.83 | 15.67 | 17.13 | 17.78 |
| 8 | 12.28±1.82 | | 9.76 | 9.91 | 10.29 | 10.75 | 11.13 | 11.90 | 13.16 | 13.97 | 14.89 | 15.73 | 16.52 |
| 9 | 12.36±1.88 | | 9.37 | 9.88 | 10.34 | 10.52 | 11.03 | 12.07 | 13.22 | 14.15 | 14.71 | 16.13 | 17.10 |
| 10 | 12.61±2.25 | | 9.43 | 9.76 | 10.26 | 10.60 | 11.00 | 12.20 | 13.91 | 14.76 | 15.16 | 17.29 | 18.21 |
| 11 | 12.26±2.25 | | 9.34 | 9.46 | 10.00 | 10.40 | 10.85 | 11.85 | 13.12 | 14.00 | 15.16 | 16.49 | 17.30 |
| 12 | 12.14±2.14 | | 9.44 | 9.50 | 9.83 | 10.25 | 10.60 | 11.72 | 13.18 | 14.09 | 14.64 | 16.70 | 17.55 |
| 13 | 12.58±1.92 | | 9.93 | 10.07 | 10.45 | 10.70 | 11.18 | 12.24 | 13.40 | 14.73 | 15.10 | 16.39 | 17.62 |
| 14 | 12.47±1.90 | | 9.75 | 10.06 | 10.44 | 10.75 | 11.20 | 12.02 | 13.55 | 14.40 | 15.20 | 15.99 | 17.03 |
| 15 | 12.57±2.13 | | 9.69 | 10.01 | 10.39 | 10.47 | 10.87 | 12.06 | 13.60 | 14.62 | 15.65 | 17.61 | 18.28 |
| 16 | 12.83±1.96 | | 9.83 | 10.02 | 10.65 | 11.03 | 11.55 | 12.59 | 13.80 | 15.04 | 15.47 | 16.99 | 17.25 |
| 17 | 13.11±2.01 | | 9.99 | 10.69 | 11.17 | 11.40 | 11.75 | 12.76 | 14.16 | 14.90 | 15.75 | 16.83 | 19.37 |
| 18 | 13.02±1.92 | | 10.25 | 10.67 | 11.15 | 11.37 | 11.74 | 12.65 | 14.05 | 14.69 | 15.61 | 16.28 | 17.57 |

**Supplementary Fig. 1.** Age- and sex-specific percentile values of the TMI in study participants aged 7–18 years.

| 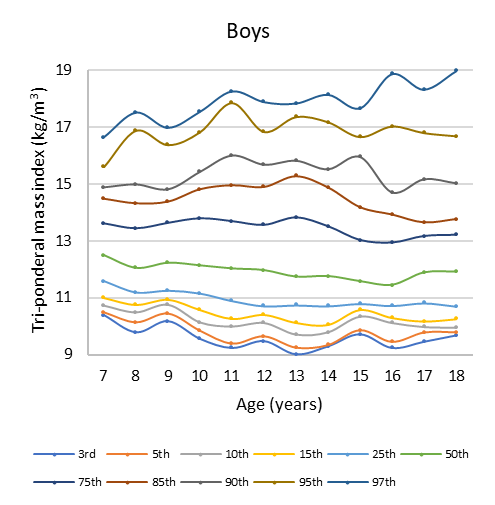 | 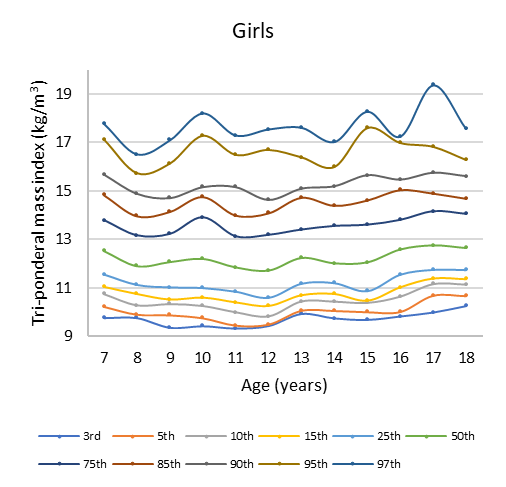 |
| --- | --- |

**Supplementary Table 2**. Age- and sex-specific percentile values of the BMI in study participants aged 7–18 years.

| **Age** | **Mean ± SD** | | **Percentiles** | | | | | | | | | | |
| --- | --- | --- | --- | --- | --- | --- | --- | --- | --- | --- | --- | --- | --- |
|  |  |  | 3rd | 5th | 10th | 15th | 25th | 50th | 75th | 85th | 90th | 95th | 97th |
| ***Boys*** |  |  |  |  |  |  |  |  |  |  |  |  |  |
| 7 | 16.18±2.27 | | 13.01 | 13.27 | 13.82 | 14.08 | 14.54 | 15.75 | 17.28 | 18.28 | 19.08 | 20.56 | 21.29 |
| 8 | 16.68±2.74 | | 13.02 | 13.58 | 13.86 | 14.26 | 14.88 | 16.07 | 17.79 | 19.22 | 20.39 | 22.76 | 23.98 |
| 9 | 17.63±2.84 | | 13.97 | 14.29 | 14.67 | 15.04 | 15.64 | 16.89 | 19.19 | 20.42 | 21.67 | 22.79 | 24.02 |
| 10 | 18.25±3.27 | | 13.84 | 14.05 | 14.63 | 15.12 | 16.00 | 17.58 | 19.98 | 21.62 | 22.51 | 25.23 | 25.91 |
| 11 | 19.10±4.07 | | 13.92 | 14.28 | 15.01 | 15.56 | 16.05 | 18.11 | 21.23 | 23.27 | 24.81 | 26.96 | 27.94 |
| 12 | 19.58±3.63 | | 14.71 | 15.06 | 15.62 | 16.13 | 16.89 | 18.76 | 21.51 | 23.43 | 25.39 | 26.97 | 27.85 |
| 13 | 20.39±4.06 | | 14.81 | 15.12 | 15.89 | 16.42 | 17.57 | 19.27 | 22.46 | 24.88 | 26.84 | 28.99 | 30.27 |
| 14 | 21.09±4.02 | | 15.62 | 16.09 | 16.99 | 17.55 | 18.22 | 20.20 | 23.19 | 24.97 | 26.49 | 29.74 | 31.50 |
| 15 | 21.11±3.15 | | 16.46 | 16.73 | 17.92 | 18.39 | 19.08 | 20.24 | 22.68 | 24.40 | 25.02 | 28.09 | 29.38 |
| 16 | 21.75±3.84 | | 17.61 | 17.82 | 18.38 | 18.59 | 19.28 | 20.60 | 22.49 | 25.16 | 26.19 | 30.33 | 34.32 |
| 17 | 22.13±3.45 | | 17.50 | 17.63 | 17.93 | 18.91 | 19.65 | 21.25 | 23.52 | 25.72 | 26.51 | 29.90 | 31.91 |
| 18 | 22.27±3.64 | | 17.60 | 17.68 | 18.17 | 18.91 | 19.94 | 21.28 | 23.55 | 25.44 | 26.37 | 30.50 | 32.30 |
| ***Girls*** | | | | | | | | | | | | | |
| 7 | 16.46±3.05 | | 12.60 | 13.05 | 13.61 | 13.99 | 14.55 | 15.74 | 17.41 | 19.64 | 20.27 | 23.56 | 23.95 |
| 8 | 16.30±2.56 | | 12.40 | 13.23 | 13.82 | 14.11 | 14.49 | 15.75 | 17.45 | 18.45 | 20.05 | 21.20 | 23.34 |
| 9 | 17.02±2.90 | | 13.19 | 13.65 | 14.15 | 14.43 | 15.02 | 16.42 | 18.23 | 19.67 | 20.98 | 23.00 | 24.19 |
| 10 | 18.28±3.55 | | 13.64 | 14.03 | 14.58 | 15.01 | 15.76 | 17.36 | 20.42 | 21.56 | 22.55 | 24.62 | 27.39 |
| 11 | 18.52±3.27 | | 14.09 | 14.28 | 15.01 | 15.51 | 16.22 | 17.89 | 20.08 | 21.54 | 22.58 | 25.55 | 27.07 |
| 12 | 19.10±3.46 | | 14.30 | 14.57 | 15.58 | 16.12 | 16.81 | 18.49 | 20.57 | 22.14 | 23.30 | 26.54 | 27.81 |
| 13 | 20.38±3.13 | | 16.03 | 16.20 | 16.86 | 17.26 | 18.07 | 19.90 | 22.06 | 23.49 | 24.68 | 26.12 | 27.83 |
| 14 | 20.55±3.12 | | 15.81 | 16.23 | 17.16 | 17.67 | 18.64 | 19.84 | 21.97 | 23.83 | 25.01 | 25.99 | 28.63 |
| 15 | 20.81±3.26 | | 16.76 | 17.01 | 17.64 | 17.86 | 18.30 | 20.08 | 21.87 | 23.96 | 26.05 | 28.13 | 29.85 |
| 16 | 21.54±3.15 | | 16.55 | 16.80 | 18.26 | 18.53 | 19.52 | 21.13 | 23.44 | 24.49 | 25.77 | 27.98 | 29.10 |
| 17 | 21.76±3.26 | | 16.12 | 17.90 | 18.81 | 19.05 | 19.33 | 21.26 | 23.39 | 24.80 | 25.85 | 27.39 | 31.39 |
| 18 | 21.69±3.18 | | 17.14 | 18.11 | 18.76 | 18.89 | 19.27 | 21.19 | 23.45 | 24.66 | 25.77 | 27.70 | 28.53 |

**Supplementary Fig. 2.** Age- and sex-specific percentile values of the BMI in study participants aged 7–18 years.

| **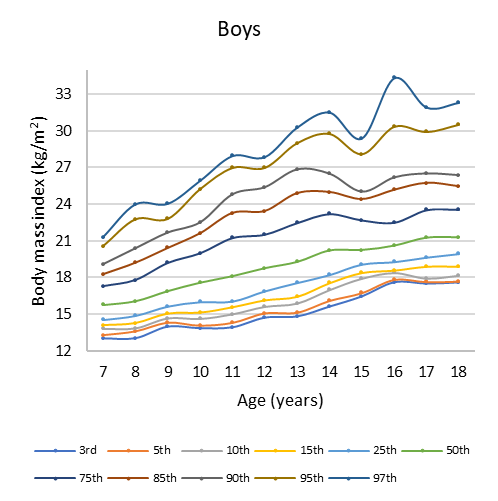** | **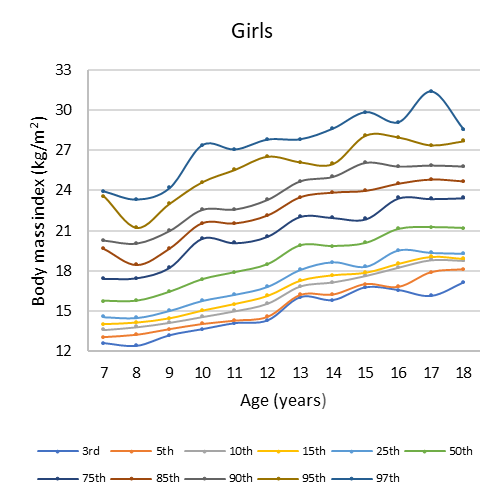** |
| --- | --- |

**Supplementary Table 3.** Characteristics of the study participants according to the quartiles of the TMI; the values are mean ± SD.

| **Variables** | **TMI quartiles** | | | |  |
| --- | --- | --- | --- | --- | --- |
|  | **1st** | **2nd** | **3rd** | **4th** | **P value** |
| ***Boys*** | N=482 | N=490 | N=492 | N=490 |  |
| Age (years) | 10.92±2.85 | 10.96±2.85 | 10.98±2.87 | 10.93±2.83 | 0.992 |
| Weight (kg) | 37.32±13.72 | 40.89±14.29 | 45.08±16.13 | 55.33±20.28 | <0.001 |
| Height (cm) | 151.87±19.24 | 150.82±18.39 | 150.64±18.33 | 151.21±17.64 | 0.820 |
| BMI (kg/m^2^) | 15.55±1.73 | 17.29±1.77 | 19.07±2.22 | 23.24±3.85 | <0.001 |
| TMI (kg/m^3^) | 10.28±0.67 | 11.50±0.42 | 12.68±0.52 | 15.37±1.78 | <0.001 |
| HC (cm) | 73.64±9.48 | 76.62±9.59 | 79.95±10.53 | 87.41±12.15 | <0.001 |
| MUAC (cm) | 20.09±2.48 | 21.19±2.68 | 22.34±2.94 | 25.11±3.45 | <0.001 |
| NC (cm) | 28.48±2.98 | 29.16±2.92 | 29.87±2.99 | 31.50±3.45 | <0.001 |
| WC (cm) | 58.87±7.05 | 61.55±7.17 | 64.38±9.07 | 74.11±12.14 | <0.001 |
| BSA | 1.25±0.30 | 1.30±0.31 | 1.36±0.33 | 1.51±0.36 | <0.001 |
| WHR | 0.80±0.06 | 0.81±0.06 | 0.81±0.06 | 0.85±0.06 | <0.001 |
| WHtR | 0.39±0.03 | 0.41±0.03 | 0.43±0.04 | 0.49±0.05 | <0.001 |
| SBP (mm Hg) | 106.46±13.63 | 109.12±13.62 | 110.78±14.05 | 115.55±14.29 | <0.001 |
| DBP (mm Hg) | 61.11±8.28 | 61.01±7.84 | 61.42±8.13 | 64.00±7.86 | <0.001 |
| MAP (mm Hg) | 76.23±8.58 | 77.05±8.13 | 77.88±8.64 | 81.18±8.69 | <0.001 |
| PP (mm Hg) | 45.35±12.36 | 48.11±12.85 | 49.36±12.59 | 51.56±12.31 | <0.001 |
| ***Girls*** | N=436 | N=437 | N=446 | N=437 |  |
| Age (years) | 10.85±2.84 | 10.87±2.85 | 10.88±2.85 | 10.86±2.84 | 0.999 |
| Weight (kg) | 35.90±11.70 | 38.21±12.14 | 42.02±13.43 | 50.94±16.17 | <0.001 |
| Height (cm) | 149.48±16.13 | 147.23±15.76 | 147.16±15.69 | 147.74±14.75 | 0.145 |
| BMI (kg/m^2^) | 15.53±1.86 | 17.05±1.87 | 18.75±2.15 | 22.66±3.62 | <0.001 |
| TMI (kg/m^3^) | 10.40±0.62 | 11.59±0.40 | 12.74±0.48 | 15.34±1.96 | <0.001 |
| HC (cm) | 73.62±9.49 | 75.66±9.98 | 78.84±10.22 | 85.17±11.82 | <0.001 |
| MUAC (cm) | 19.76±2.23 | 20.53±2.20 | 21.81±2.48 | 24.16±2.96 | <0.001 |
| NC (cm) | 27.11±2.31 | 27.59±2.25 | 28.20±2.21 | 29.68±2.48 | <0.001 |
| WC (cm) | 56.26±5.42 | 57.97±5.80 | 60.98±6.37 | 69.01±10.07 | <0.001 |
| BSA | 1.21±0.26 | 1.24±0.26 | 1.30±0.28 | 1.44±0.30 | <0.001 |
| WHR | 0.77±0.07 | 0.77±0.06 | 0.78±0.06 | 0.81±0.07 | <0.001 |
| WHtR | 0.38±0.03 | 0.40±0.03 | 0.42±0.03 | 0.47±0.06 | <0.001 |
| SBP (mm Hg) | 104.31±11.38 | 106.50±11.77 | 108.74±12.18 | 111.97±13.46 | <0.001 |
| DBP (mm Hg) | 61.69±8.02 | 62.42±7.92 | 62.63±8.13 | 65.18±8.30 | <0.001 |
| MAP (mm Hg) | 75.90±7.99 | 77.11±8.06 | 78.00±8.36 | 80.77±8.89 | <0.001 |
| PP (mm Hg) | 42.62±10.00 | 44.09±10.17 | 46.12±10.30 | 46.79±11.08 | <0.001 |

BMI – body mass index, TMI – tri‑ponderal mass index, HC – hip circumference, MUAC – mid-upper arm circumference, NC – neck circumference, WC – waist circumference, BSA – body surface area, WHR – waist-hip ratio, WHtR – waist-to-height ratio, SBP – systolic blood pressure, DBP – diastolic blood pressure, MAP – mean arterial pressure, PP – pulse pressure.

Values are numbers (percentages) and mean ± SD (standard deviation).

**Supplementary Table 4.** Characteristics of the study participants according to the quartiles of the BMI; the values are mean ± SD.

| **Variables** | **BMI quartiles** | | | | **P value** |
| --- | --- | --- | --- | --- | --- |
|  | **1st** | **2nd** | **3rd** | **4th** |  |
| ***Boys*** | N=482 | N=483 | N=498 | N=491 |  |
| Age (years) | 10.95±2.84 | 10.99±2.84 | 10.92±2.87 | 10.93±2.84 | 0.971 |
| Weight (kg) | 35.17±12.39 | 40.78±14.09 | 45.36±15.06 | 57.18±20.16 | <0.001 |
| Height (cm) | 148.52±18.62 | 150.93±18.79 | 151.51±18.04 | 153.52±17.87 | <0.001 |
| BMI (kg/m^2^) | 15.38±1.59 | 17.26±1.65 | 19.08±1.20 | 23.39±3.75 | <0.001 |
| TMI (kg/m^3^) | 10.41±0.80 | 11.49±0.64 | 12.64±0.78 | 15.26±1.89 | <0.001 |
| HC (cm) | 72.66±9.15 | 76.65±9.60 | 79.94±9.84 | 88.30±11.85 | <0.001 |
| MUAC (cm) | 19.85±2.41 | 21.17±2.58 | 22.36±2.81 | 25.32±3.33 | <0.001 |
| NC (cm) | 28.20±2.87 | 29.12±2.91 | 29.98±2.88 | 31.68±3.42 | <0.001 |
| WC (cm) | 58.20±6.55 | 61.27±7.12 | 64.56±8.61 | 74.79±11.81 | <0.001 |
| BSA | 1.20±0.28 | 1.30±0.30 | 1.37±0.31 | 1.55±0.36 | <0.001 |
| WHR | 0.80±0.06 | 0.80±0.05 | 0.81±0.05 | 0.85±0.06 | <0.001 |
| WHtR | 0.39±0.03 | 0.41±0.03 | 0.43±0.04 | 0.49±0.06 | <0.001 |
| SBP (mm Hg) | 105.42±13.43 | 108.79±13.77 | 111.45±13.65 | 116.19±14.09 | <0.001 |
| DBP (mm Hg) | 60.69±8.24 | 60.65±7.85 | 61.94±8.14 | 64.23±7.72 | <0.001 |
| MAP (mm Hg) | 75.60±8.55 | 76.70±8.11 | 78.45±8.50 | 81.55±8.53 | <0.001 |
| PP (mm Hg) | 44.72±12.04 | 48.15±13.16 | 49.51±12.40 | 51.96±12.21 | <0.001 |
| ***Girls*** | N=434 | N=438 | N=446 | N=438 |  |
| Age (years) | 10.85±2.85 | 10.89±2.85 | 10.87±2.85 | 10.86±2.84 | 0.998 |
| Weight (kg) | 33.35±10.46 | 38.43±12.08 | 42.45±12.68 | 52.75±15.43 | <0.001 |
| Height (cm) | 145.43±15.56 | 147.63±16.54 | 148.25±15.54 | 150.24±14.37 | <0.001 |
| BMI (kg/m^2^) | 15.30±1.72 | 17.09±1.74 | 18.76±1.99 | 22.83±3.46 | <0.001 |
| TMI (kg/m^3^) | 10.54±0.75 | 11.61±0.69 | 12.68±0.76 | 15.22±2.06 | <0.001 |
| HC (cm) | 71.95±9.15 | 75.93±9.70 | 79.02±9.63 | 86.34±11.36 | <0.001 |
| MUAC (cm) | 19.44±2.23 | 20.67±2.07 | 21.77±2.32 | 24.38±2.82 | <0.001 |
| NC (cm) | 26.74±2.20 | 27.70±2.18 | 28.27±2.14 | 29.87±2.41 | <0.001 |
| WC (cm) | 55.58±5.26 | 58.03±5.46 | 60.93±5.96 | 69.63±9.74 | <0.001 |
| BSA | 1.15±0.24 | 1.25±0.27 | 1.32±0.27 | 1.48±0.28 | <0.001 |
| WHR | 0.78±0.07 | 0.77±0.06 | 0.78±0.06 | 0.81±0.08 | <0.001 |
| WHtR | 0.38±0.03 | 0.40±0.03 | 0.41±0.04 | 0.46±0.06 | <0.001 |
| SBP (mm Hg) | 103.58±11.62 | 106.72±11.61 | 107.89±11.90 | 113.32±12.99 | <0.001 |
| DBP (mm Hg) | 61.30±7.84 | 62.31±8.21 | 62.46±8.04 | 65.83±7.99 | <0.001 |
| MAP (mm Hg) | 75.40±7.94 | 77.11±8.22 | 77.60±8.16 | 81.66±8.53 | <0.001 |
| PP (mm Hg) | 42.28±10.18 | 44.41±10.04 | 45.43±10.34 | 47.49±10.85 | <0.001 |

BMI – body mass index, TMI – tri‑ponderal mass index, HC – hip circumference, MUAC – mid-upper arm circumference, NC – neck circumference, WC – waist circumference, BSA – body surface area, WHR – waist-hip ratio, WHtR – waist-to-height ratio, SBP – systolic blood pressure, DBP – diastolic blood pressure, MAP – mean arterial pressure, PP – pulse pressure.

Values are numbers (percentages) and mean ± SD (standard deviation).

**Supplementary Table 5.** Characteristics of the study participants according to age- and sex-specific TMI percentile categories; the values are mean ± SD.

| **Variables** | **Underweight/Normal weight** | **Overweight** | **Obesity** | **P value** |
| --- | --- | --- | --- | --- |
| ***Boys*** | N=1664 | N=197 | N=93 |  |
| Age (years) | 10.95±2.85 | 11.01±2.87 | 10.85±2.81 | 0.923 |
| Weight (kg) | 42.17±15.65 | 56.09±19.28 | 65.55±23.63 | <0.001 |
| Height (cm) | 151.04±18.59 | 151.86±17.11 | 151.19±17.80 | 0.622 |
| BMI (kg/m^2^) | 17.77±2.71 | 23.40±3.04 | 27.53±4.11 | <0.001 |
| TMI (kg/m^3^) | 11.80±1.34 | 15.40±0.81 | 18.21±1.72 | <0.001 |
| HC (cm) | 77.64±10.60 | 87.71±11.55 | 93.89±12.82 | <0.001 |
| MUAC (cm) | 21.54±3.02 | 25.41±3.32 | 27.01±3.44 | <0.001 |
| NC (cm) | 29.36±3.08 | 31.65±3.40 | 32.92±3.48 | <0.001 |
| WC (cm) | 62.61±8.81 | 74.26±11.27 | 82.81±13.40 | <0.001 |
| BSA | 1.32±0.32 | 1.53±0.35 | 1.65±0.39 | <0.001 |
| WHR | 0.81±0.06 | 0.85±0.05 | 0.88±0.06 | <0.001 |
| WHtR | 0.42±0.04 | 0.49±0.04 | 0.55±0.05 | <0.001 |
| SBP (mm Hg) | 109.39±13.97 | 116.04±14.64 | 118.59±13.84 | <0.001 |
| DBP (mm Hg) | 61.42±8.12 | 64.02±7.54 | 65.82±7.46 | <0.001 |
| MAP (mm Hg) | 77.41±8.55 | 81.36±8.74 | 83.41±8.19 | <0.001 |
| PP (mm Hg) | 47.97±12.70 | 52.02±12.17 | 52.77±12.36 | <0.001 |
| ***Girls*** | N=1488 | N=182 | N=86 |  |
| Age (years) | 10.85±2.85 | 11.00±2.82 | 10.85±2.90 | 0.735 |
| Weight (kg) | 39.37±12.89 | 51.98±14.79 | 61.74±17.79 | <0.001 |
| Height (cm) | 147.70±15.74 | 149.15±14.67 | 148.71±15.15 | 0.286 |
| BMI (kg/m^2^) | 17.48±2.55 | 22.73±2.48 | 27.22±3.50 | <0.001 |
| TMI (kg/m^3^) | 11.85±1.27 | 15.24±0.78 | 18.36±2.13 | <0.001 |
| HC (cm) | 76.56±10.16 | 86.14±11.58 | 92.44±12.23 | <0.001 |
| MUAC (cm) | 20.98±2.59 | 24.05±2.61 | 26.53±2.90 | <0.001 |
| NC (cm) | 27.77±2.30 | 29.72±2.39 | 31.44±2.43 | <0.001 |
| WC (cm) | 59.04±6.55 | 69.51±9.03 | 78.03±10.36 | <0.001 |
| BSA | 1.26±0.27 | 1.46±0.28 | 1.59±0.31 | <0.001 |
| WHR | 0.78±0.07 | 0.81±0.07 | 0.85±0.08 | <0.001 |
| WHtR | 0.40±0.04 | 0.47±0.05 | 0.53±0.05 | <0.001 |
| SBP (mm Hg) | 106.83±11.99 | 111.74±13.61 | 117.99±13.55 | <0.001 |
| DBP (mm Hg) | 62.43±8.10 | 65.02±8.00 | 68.15±8.00 | <0.001 |
| MAP (mm Hg) | 77.23±8.25 | 80.59±8.71 | 84.76±8.64 | <0.001 |
| PP (mm Hg) | 44.41±10.28 | 46.71±11.33 | 49.84±11.24 | <0.001 |

BMI – body mass index, TMI – tri‑ponderal mass index, HC – hip circumference, MUAC – mid-upper arm circumference, NC – neck circumference, WC – waist circumference, BSA – body surface area, WHR – waist-hip ratio, WHtR – waist-to-height ratio, SBP – systolic blood pressure, DBP – diastolic blood pressure, MAP – mean arterial pressure, PP – pulse pressure.

Values are numbers (percentages) and mean ± SD (standard deviation).

**Supplementary Table 6.** Characteristics of the study participants according to the BMI categories (based on the IOTF criteria); the values are mean ± SD.

| **Variables** | **Normal weight** | **Overweight** | **Obesity** | **P value** |
| --- | --- | --- | --- | --- |
| ***Boys*** | N=1474 | N=346 | N=134 |  |
| Age (years) | 10.89±2.92 | 11.12±2.62 | 11.13±2.63 | 0.08 |
| Weight (kg) | 40.23±14.68 | 54.61±16.65 | 68.03±21.04 | <0.001 |
| Height (cm) | 149.95±18.80 | 154.47±16.81 | 155.49±16.22 | <0.001 |
| BMI (kg/m^2^) | 17.22±2.28 | 22.23±2.24 | 27.32±3.58 | <0.001 |
| TMI (kg/m^3^) | 11.54±1.18 | 14.44±0.95 | 17.60±1.74 | <0.001 |
| HC (cm) | 76.24±10.00 | 87.04±9.98 | 94.88±11.46 | <0.001 |
| MUAC (cm) | 21.09±2.79 | 24.93±2.80 | 27.28±3.13 | <0.001 |
| NC (cm) | 29.08±3.01 | 31.32±3.07 | 33.20±3.21 | <0.001 |
| WC (cm) | 61.23±7.81 | 72.64±9.69 | 83.04±12.10 | <0.001 |
| BSA | 1.29±0.31 | 1.52±0.31 | 1.70±0.35 | <0.001 |
| WHR | 0.81±0.06 | 0.83±0.05 | 0.87±0.06 | <0.001 |
| WHtR | 0.41±0.04 | 0.47±0.05 | 0.53±0.06 | <0.001 |
| SBP (mm Hg) | 108.40±13.81 | 115.63±13.70 | 120.35±13.39 | <0.001 |
| DBP (mm Hg) | 60.99±8.05 | 64.04±7.93 | 66.24±6.85 | <0.001 |
| MAP (mm Hg) | 76.79±8.43 | 81.24±8.50 | 84.27±7.63 | <0.001 |
| PP (mm Hg) | 47.41±12.68 | 51.59±12.05 | 54.11±12.16 | <0.001 |
| ***Girls*** | N=1416 | N=258 | N=82 |  |
| Age (years) | 10.93±2.87 | 10.81±2.72 | 9.98±2.63 | 0.011 |
| Weight (kg) | 38.96±12.87 | 51.36±14.27 | 60.07±18.03 | <0.001 |
| Height (cm) | 147.57±15.96 | 149.86±13.62 | 147.34±15.01 | 0.08 |
| BMI (kg/m^2^) | 17.32±2.49 | 22.32±2.58 | 26.96±3.64 | <0.001 |
| TMI (kg/m^3^) | 11.75±1.21 | 14.89±1.04 | 18.35±2.22 | <0.001 |
| HC (cm) | 76.30±10.21 | 85.57±10.93 | 90.62±12.87 | <0.001 |
| MUAC (cm) | 20.82±2.49 | 24.26±2.69 | 26.05±3.04 | <0.001 |
| NC (cm) | 27.70±2.31 | 29.62±2.25 | 31.18±2.63 | <0.001 |
| WC (cm) | 58.64±6.29 | 69.05±8.62 | 77.59±10.76 | <0.001 |
| BSA | 1.26±0.27 | 1.46±0.27 | 1.56±0.31 | <0.001 |
| WHR | 0.77±0.06 | 0.81±0.07 | 0.86±0.08 | <0.001 |
| WHtR | 0.40±0.04 | 0.46±0.05 | 0.53±0.05 | <0.001 |
| SBP (mm Hg) | 106.51±11.92 | 113.27±13.62 | 114.79±12.66 | <0.001 |
| DBP (mm Hg) | 62.37±8.14 | 64.99±8.07 | 67.13±7.37 | <0.001 |
| MAP (mm Hg) | 77.08±8.27 | 81.09±8.77 | 83.02±8.03 | <0.001 |
| PP (mm Hg) | 44.14±10.22 | 48.28±11.27 | 47.66±10.64 | <0.001 |

BMI – body mass index, TMI – tri‑ponderal mass index, HC – hip circumference, MUAC – mid-upper arm circumference, NC – neck circumference, WC – waist circumference, BSA – body surface area, WHR – waist-hip ratio, WHtR – waist-to-height ratio, SBP – systolic blood pressure, DBP – diastolic blood pressure, MAP – mean arterial pressure, PP – pulse pressure.

Values are numbers (percentages) and mean ± SD (standard deviation).

Supplementary Fig. 3. Area under ROC curves of anthropometric indices to predict HBP.

| 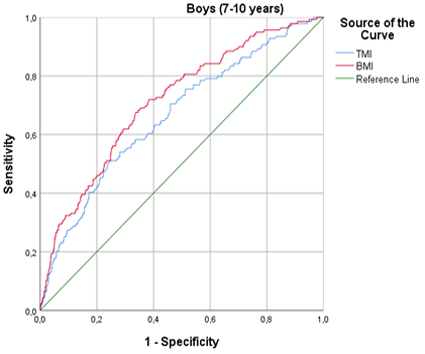 | 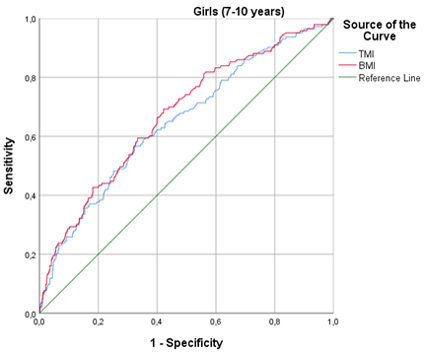 |
| --- | --- |
|  |  |
| 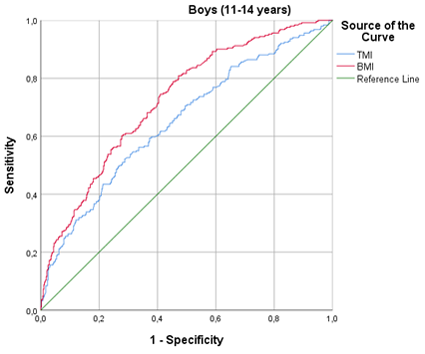 | 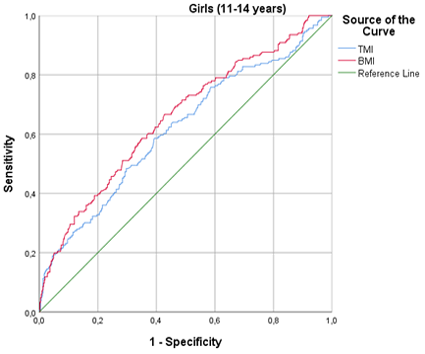 |
|  |  |
| 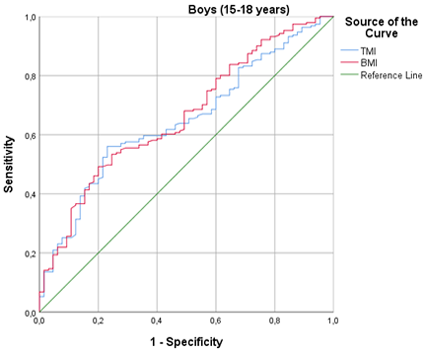 | 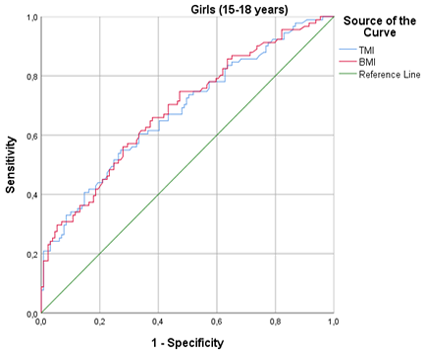 |
|  |  |
